# Increased longevity of circulating human IgG in an NSG Fc gamma receptor-1 deficient humanized mouse model

**DOI:** 10.1016/j.xphs.2025.103964

**Published:** 2025-08-16

**Authors:** Hannah R. Megathlin, Lisa Burzenski, Michael A. Brehm, Dale L. Greiner, Sathy Balu-Iyer, Leonard D. Shultz

**Affiliations:** a The Jackson Laboratory, Bar Harbor, Maine 04609, USA; b Graduate School of Biomedical Sciences and Engineering, University of Maine, Orono, ME 04469, USA; c Program in Molecular Medicine, Diabetes Center of Excellence, UMass Chan Medical School, Worcester, MA 01605, USA; d Department of Pharmaceutical Sciences, University of Buffalo, The State University of New York, Buffalo, NY 14261, USA

**Keywords:** Monoclonal antibody(s), Igg antibody(s), Pharmacodynamics, Cancer, Immunotherapy

## Abstract

Monoclonal antibodies (mAbs) are powerful therapeutic tools that are used to treat multiple types of human cancer as well as a diverse set of non-malignant diseases. Humanized NOD.Cg-*Prkdc*^*scid*^ *Il2rgt*^*m1Wjl*^/SzJ (NSG) mice implanted with human tumors and with immune cells and tissues are widely used in studies of mAb-based therapeutics. However, due to a gain of function mutation in the *Fcgr1* gene in the NOD strain background, NSG mice rapidly clear human IgG1, IgG3, and IgG4. As most mAbs are either IgG1 or IgG4 isotypes, the use of NOD-based mouse models for preclinical testing of therapeutic mAbs is limited by the reduced half-life in vivo. In order to extend the half-life of mAbs in NSG mice and create a more physiologically relevant model, we created NSG Fcγ Receptor I knock out (NSG-*Fcgr1*^*null*^) mice. IgG clearance was measured for three different cancer therapeutic mAbs: rituximab (IgG1), trastuzumab (IgG1), and pembrolizumab (IgG4), by comparing the levels of circulating human IgG over the course of 5 weeks post IV injection in NSG and NSG-*Fcgr1*^*null*^ mice. Preliminary pharmacokinetic analyses found significant increases in the half-lives and exposure of each of these mAbs in the NSG-*Fcgr1*^*null*^ mice when compared to NSG controls. Additionally, when engrafted with human hematopoietic stem cells (HSCs), NSG-*Fcgr1*^*null*^ mice supported higher levels of serum IgG when compared to NSG controls. Overall, the NSG-*Fcgr1*^*null*^ mouse presents a more physiologically relevant and translatable model for the in vivo testing of human therapeutic mAbs.

## Introduction

Monoclonal antibodies (mAbs) have emerged as a revolutionary therapeutic tool for human disease. mAb-based therapeutics are currently being used to treat a wide variety of diseases, including cancer,^1,2^ autoimmune diseases,^3–7^ cardiovascular disease, and neurological disorders.^8^ Humanized mice are widely used for testing the preclinical efficacy of human mAbs in vivo. Engraftment of severely immunodeficient mice with hematopoietic stem cells (HSCs) or human hematolymphoid cells or tissues supports development of functional human immune systems. NOD.Cg-*Prkdc*^*scid*^ *Il2rg*^*m1Wjl*^/SzJ (NSG) mice, which lack mature B cells, T cells, and natural killer (NK) cells, are the most widely used model for human hematopoietic engraftment.^9^ NSG mice lack a functional IL2 common receptor γ-chain (IL2RG), preventing signaling through six different cytokine receptors (IL2, IL4, IL7, IL9, IL15, and IL21) that require IL2RG function.^10^ In addition NSG mice have a human-like polymorphism at the signal regulatory alpha *(Sirpa)* locus^11^ that protects engrafted human cells from phagocytosis by mouse macrophages. NSG mice support high levels of human HSC engraftment and develop human lymphoid and myeloid cells post-engraftment. Human HSC and peripheral blood mononuclear cell (PBMC) engrafted NSG mice are widely used tools for developing and validating preclinical treatments for human diseases.^12^

Due to increasing interest in mAb therapy for a spectrum of human diseases, there is a significant need for humanized mouse models that can effectively model the pharmacokinetics and function of these therapeutic mAbs in vivo. Although NSG mice are commonly used for mAb functional validation, non-obese diabetic (NOD) mice, the background strain mice for NSG mice, have a gain of function mutation in *Fcgr1* gene, which encodes the FcγRI protein and significantly reduces human IgG half-life in the circulation. FcγRs bind to IgG, the most abundant antibody class in circulation. Both mice and humans have four subclasses of IgG,^13^ but there are nomenclature differences between mice and humans.^14^ Human IgG mAbs bind to multiple mouse Fc gamma receptors (FcγRs): FcγRI, FcγRIIb, FcγRIII, and FcγRIV. Mouse FcγRI (mFcγRI) is the only murine FcγR that binds with high affinity to human IgG1 and IgG4^14^.

Therapeutic mAbs isotypes are predominantly either IgG1, IgG2, or IgG4,^15^ with IgG1 isotype being the most common.^16^ The half-life of human IgG1 in NSG mice is 1.4 days compared to a half-life of 16 days for C.B17-*scid* mice.^17^ We tested the hypothesis that eliminating the activity of FcγRI in NSG mice would increase the half-life of therapeutic human IgG mAbs in vivo and enhance preclinical testing of human mAbs.^18^ In this study, we describe the generation of a new NSG mouse model that lacks mouse FcγRI and supports human HSC engraftment. The *Fcgr1* gene was targeted for genetic editing in NSG mice, and this new mouse model, NOD.Cg-*Fcgr1*^*em1Sz*^ *Prkdc*^*scid*^ *Il2rg*^*tm1Wjl*^/Sz (NSG-*Fcgr1*^*null*^), recapitulates the in vivo clearance of human IgG1, IgG3, and IgG4 mAbs observed in humans. The NSG-*Fcgr1*^*null*^ mouse will be a useful model for preclinical testing of therapeutic human mAbs.

## Methods

### Mice

All experiments were done under the approval of the Animal Care and Use Committee of The Jackson Laboratory. The mice were housed in high barrier pathogen-free housing at bio-safety level 2 status with a 12-hour light/12-hour dark cycle at 68–72°F temperature.

NSG-*Fcgr1*^*null*^ mice were generated using CRISPR-Cas9. The upstream RNA guides used were: 5′-TCTGGGTACCGAAAGGCGGG-3′ and 5′-TCCCAATAAAACTTCACCAG-3′. The downstream guides used were: 5′-GGATCCGGCTGAGACAAGCT-3′ and 5′-ATGGAGTCAGGTCACAGCGG-3′. Guides, at a concentration of 300 ng/μL, and the Cas9 protein (Integrated DNA Technologies, Inc., Coralville, IA) at a concentration of 500 ng/μL, were electroporated into the zygotes as previously published.^19^ The mice with the largest 4 deletions were chosen to establish lines, and ultimately the line with an 8622 bp deletion beginning at 1355 was chosen for expansion and fixation to homozygosity. Removal of exons 3–5 and part of exon 6 in the *Fcgr1* gene was confirmed through Sanger sequencing.^20^

### Polymerase chain reaction (PCR)

To confirm genotyping of NSG-*Fcgr1*^*null*^ mice, the following primers were used: KO forward 5′-CCAATGGAGAAGTGGATGGA-3′, KO reverse 5′-ATTCCGATGCTCTCAGGATG-3′, WT forward 5′-GCTGACTTGGGGTGGCTACTAA-3′, and WT reverse 5′-ATCTTCTCTTTCCGCCCTTGCT-3′, purchased from IDT (Integrated DNA Technologies, Inc., Coralville, IA). DNA was prepared using the Qiagen DNeasy kit (Qiagen USA, Germantown, MD) and the PCR was performed by combining primers with Taq DNA polymerase (New England Biolabs Inc., Ipswich, MA), cresol red, deoxynucleotides, and nuclease-free water and cycled on a C1000 Touch Thermocycler (Bio-Rad Laboratories, Inc., Hercules, CA) and run out on a 2 % agarose gel.^21^

### RNA isolation and RT-PCR

RNA was isolated from livers of NSG and NSG-*Fcgr1*^*null*^ mice using New England Biolabs Monarch^®^ Total RNA Miniprep Kit as described,^22^ following manufacturer instructions. Briefly, liver samples were homogenized in DNA/RNA Protection Reagent. Protein was removed using Proteinase K (New England Biolabs Inc., Ipswich, MA), and samples were incubated for 5 min at 55 °C. Samples were lysed using RNA Lysis buffer (New England Biolabs Inc., Ipswich, MA) and spun through provided columns to remove genomic DNA. Equal volume of 70 % Ethanol was added. Samples were run through RNA purification columns provided and RNA was eluted with nuclease free water. RNA concentration and purity was measured using a NanoDrop 1000 (Thermofisher Scientific Inc., Waltham, MA). RT-PCR was performed using the New England Biolabs One*Taq*^®^ RT-PCR Kit, following manufacturer instructions. Briefly, RNA was combined with d(T)_23_VN and RNA was denatured for 5 min at 70 °C. M-MuLV Enzyme mix was added to samples and were incubated for 1 hour at 42 °C to form the first strand of cDNA.^22^ To amplify the cDNA, One*Taq* Hot Start Master Mix was used with the following primers: forward 5′-CGATGGCGTGTATGAAGAAGTA-3′ and reverse 5′-AGGGAGGTTAGATGGATGGATC-3 (Integrated DNA Technologies (Coraville, IA).

### IgG clearance using therapeutic mAbs

*Rituximab* (Rituxan, 10 mg/mL, Genentech), is an IgG1k mouse chimeric murine/human mAb directed against human CD20 is used to treat B cell malignancies and other human diseases. Cohorts of 5 female and 5 male NSG-*Fcgr1*^*null*^ mice and NSG controls at 5 weeks of age were injected IV by tail vein with 200 μL of 1 mg/mL rituximab for a total dose of 200 μg per mouse. Blood was collected in serum separator tubes from the submental vein^23^ at 24 h, 48 h, 5 days, 1 week post rituximab injection, and then weekly for 5 weeks. Blood was spun in an Eppendorf 5418R refrigerated centrifuge at 10,000 g for 10 min and serum was stored at −80C°.

*Trastuzumab* (Herceptin, 21 mg/mL, Genentech,) an IgG1k monoclonal mouse chimeric murine/human mAb that targets human epidermal growth factor receptor 2 (HER2). Cohorts of 5 female NSG-*Fcgr1*^*null*^ mice and NSG controls at 9-weeks of age were injected IV by tail vein with 100 μL of 2.1 mg/mL for a total dose of 210 μg per mouse. Blood was collected by submental vein in serum separator tubes at 1 hour, 24 h, 48 h, 1 week and then weekly for 5 weeks post-injection and stored as described above.

*Pembrolizumab* (Keytruda, 25 mg/mL, Merck) a human IgG4k mAb that targets human programmed death-protein 1 (PD-1). Cohorts of 5 females of NSG-*Fcgr1*^*null*^ mice and NSG controls at 5 weeks of age were each given 200 μL of 0.1 mg/mL for a total dose of 20 μg per mouse. Serum was collected as described above at 2 h, 24 h, 48 h, 5 days, 1 week, and then weekly for 5 weeks post injection and stored as described above.

### IgG ELISA

Serum IgG levels were determined by enzyme-linked immunosorbent assay (ELISA). The ELISA kits were purchased from Fortis Life Sciences., Montgomery, TX. Briefly, 96-well plates were coated overnight at 4 C using purified goat anti-human IgG (catalog # A80–104A Fortis Life Sciences, Montgomery, TX) diluted 1 to 100. The plates were washed with TBS-tween and then blocked using 10 % bovine serum albumin in TBS for 30 min at room temperature. Plates were washed and samples were diluted at both 1 to 200 and 1 to 2000. Samples and standards were added and were incubated for 1 hour at room temperature. After washing, horseradish peroxidase conjugated goat anti-human IgG (Fortis Life Sciences., Montgomery, TX) diluted 1 to 100,000 was applied and then incubated at room temperature for 1 hour. Substrate solution (R&D Systems, Inc., Minneapolis, MN). 3,3′, 5,5′ tetramethylbenzidine dihydrochloride (TMB) substrate was prepared according to manufacturer’s instructions and added to the wells. The reaction was developed for 15 min in the dark before 0.18 M sulfuric acid was used to stop the reaction. The concentration of each well was determined using a Spectramax i3 (Molecular Devices, LLC., San Jose, CA) which measured the optical density values and converted those to concentrations based on the standard curve. Significance between curves was determined using RM-ANOVA on GraphPad Prism GraphPad Prism 10.1.0 (GraphPad Software Inc., La Jolla, CA). The standards used and their concentrations for each experiment are listed below.

Standards of rituximab (maximum of 500 ng/mL to a minimum of 7.8 ng/mL) were used.

Standards of trastuzumab (maximum of 500 ng/mL to a minimum of 7.8 ng/mL) were used.

Standards of pembrolizumab (maximum concentration of 1000 ng/mL to a minimum of 7.8 ng/mL) were used.

*Human IgG from HSC engrafted mice.* For quantification of human IgG in mice engrafted with human HSCs (see below), ELISAs were carried out as above and human IgG (Fortis Life Sciences) was used as a standard (maximum of 500 ng/mL to a minimum of 7.8 ng/mL).

### Human HSC isolation and engraftment of mice

Human umbilical cord blood (UCB) was obtained in accordance with the Committee for the Protection of Human Subjects in Research guidelines of the University of Massachusetts Chan Medical School. UCB was provided by the medical staff of the University of Massachusetts Memorial Umbilical Cord Blood Donation Program as deidentified specimens. Groups 5 female NSG and NSG-*Fcgr1*^*null*^ mice at 8-weeks of age were irradiated with 200 cGy^24^ with an X-RAD320. Irradiated mice were injected intravenously (IV) with 1.5 × 10^5^ CD34+ human HSC.^25,26^ Following cell transfer, mice were maintained in a biosafety level 2 mouse room as described above.

### Flow cytometry

Phenotyping of leukocyte subpopulations in lymphoid organs and bone marrow was conducted as follows. Briefly, spleens were excised and placed in cold flow cytometry buffer (PBS containing 5 % fetal bovine sera (FBS) and 0.1 % sodium azide). The spleens were then pressed through sterile 70 μm nylon mesh cell strainers (Asheville, NC) to make single cell suspensions. Bone marrow cells were harvested by removing both femurs and crushing them with a mortar and pestle, then filtering through 70 μm cell strainers. Erythrocytes were lysed in buffered ammonium chloride. Nucleated cells were washed twice with flow cytometry buffer. To block nonspecific binding, all suspensions were incubated for 60 min with 1 mg/mL rabbit IgG (Sigma Chemical Co., St. Louis, MO) in 100 μL total volume. All incubations with antibodies were conducted for 60 min on ice prior to being washed and resuspended in 500 μL of PBS containing 0.1 % sodium azide and 3 μM propidium iodide. Viable cells were analyzed for cell surface differentiation markers on a Attune NxT Flow Cytometer (Thermofisher Scientific Inc., Waltham, MA). Cytometric analyses were conducted on 1 × 10^4^ to 1 × 10^6^ viable leukocytes. Nucleated cells were selected by light scatter. Nonviable cells were gated out on the basis of propidium iodide staining. Statistics were run using GraphPad Prism 10.1.0 (GraphPad Software Inc., La Jolla, CA).

### Pharmacokinetics

Noncompartmental pharmacokinetic analysis was performed using the PKNCA package for R.^27^ The parameters assessed were the area under the curve to infinity (AUC_Inf_), apparent terminal rate constant (lambda.z), volume of distribution (Vd), and clearance (CL). The AUC_Inf_ was determined using the linear method for all three mAbs.

### Keytruda functional study

For human immune system engraftment, both female and male NSG and NSG-*Fcgr1*^*null*^ mice were irradiated with 200 cGy using a Cesium-137 irradiator (GammaCell 40 Exactor, Best Theratronics, Ottawa, Canada) between 6 and 10 weeks of age and 4 h later, injected IV with 5 × 10^4^ CD34+ HSC prepared as described above. Human immune system engraftment was confirmed by flow cytometry 16 weeks later, and mice with greater than 25 % human CD45+ cells and greater than 10 % CD3+ *T* cells (as a % of human CD45) in the peripheral blood were used in experiments. HSC engrafted NSG and NSG-*Fcgr1*^*null*^ mice were implanted with a patient derived melanoma that was obtained from the UMass Chan Medical School Cancer Avatar Institute (IRB ID: H00004721) The tumor was passaged in NSG mice 4 times to deplete the human leukocytes present within the primary tumor microenvironment. For tumor implant, the patient derived xenograft (PDX) melanoma was processed into a single cell suspension and 2.5 × 10^6^ cells were transplanted subcutaneously to the right flank of the mice. The mice were monitored for tumor growth and the indicated mice were treated IV with pembrolizumab (MERCK) or an IgG4 isotype control (BioXcell, Lebanon, NH) (5 mg/kg) every 3 to 4 days for a total of 5 injections starting when the tumors were between 25 and 100 mm^3^ in volume. Tumor volumes were monitored by caliper measurement described^22^ over the course of the experiment. Significance between curves was determined using RM-ANOVA on GraphPad Prism GraphPad Prism 10.1.0 (GraphPad Software Inc., La Jolla, CA).

## Results

### Mouse Fc receptor and human IgG binding

Mouse Fc receptors are capable of binding both mouse and human IgGs and share names and CD numbers with human Fc receptors. Mouse Fc receptors are more readily able to bind to human IgG isotypes than human Fc receptors are able to bind mouse IgG isotypes.^28^ Human IgG isotypes bind to mouse Fc receptors with varying affinities (Supplemental Fig. 1).

### Generation and phenotypic characterization of NSG-Fcgr1^null^ mice

**Fig. 1.**
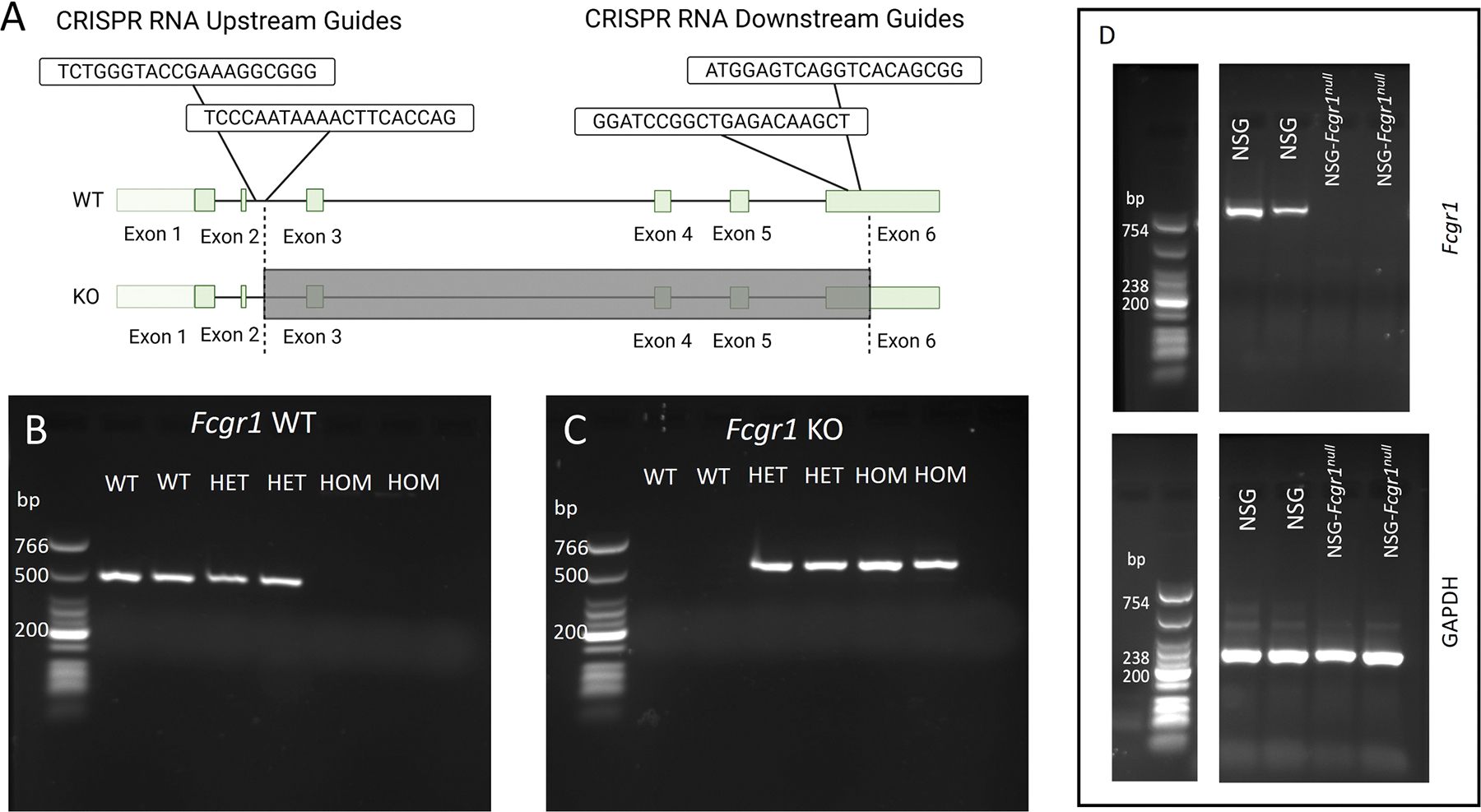
Generation of NSG-*Fcgr1*^*null*^ mice. (A) *Fcgr1* wild type (WT) gene with areas removed using CRISPR-Cas9 in the knockout (KO) homozygous (HOM) mice shown in dark grey. (B & C) Gel electrophoresis of polymerase chain reaction (PCR) for FcγRI WT (B) and KO (C) alleles with expected products of 445 and 485 bp, respectively. (D) RT-PCR showing the absence of Fcgr1 expression in the liver of the NSG-*Fcgr1*^*null*^ mice. The top image is for the *Fcgr1* gene, with an expected product of 754 bp. The bottom image illustrates the presence of GAPDH Primers for GAPDH were run at the same time using the same samples to establish the presence of RNA.

The CRISPR-Cas9^29^ (Fig. 1A) approach yielded 19 founder mice with deletions in the targeted area. PCR and gel electrophoresis demonstrated no bands at 445 bp for the wild type allele bands at 485 bp for the knockout allele in the NSG-*Fcgr1*^*null*^ mice (Fig. 1B–C). We were unable to demonstrate specific staining of FcγRI in NSG or NOD mice using commercially available anti-mouse FcγRI antibodies by flow cytometry or histochemistry, due to the NOD mouse’s *FcgR1* d allele not reacting with commercial antibodies.^30^ Ultimately, we performed reverse transcription PCR (RT-PCR) to confirm that no FcγRI RNA was being produced by NSG-*Fcgr1*^*null*^ mice (Fig. 1D).

### mAb clearance

**Fig. 2.**
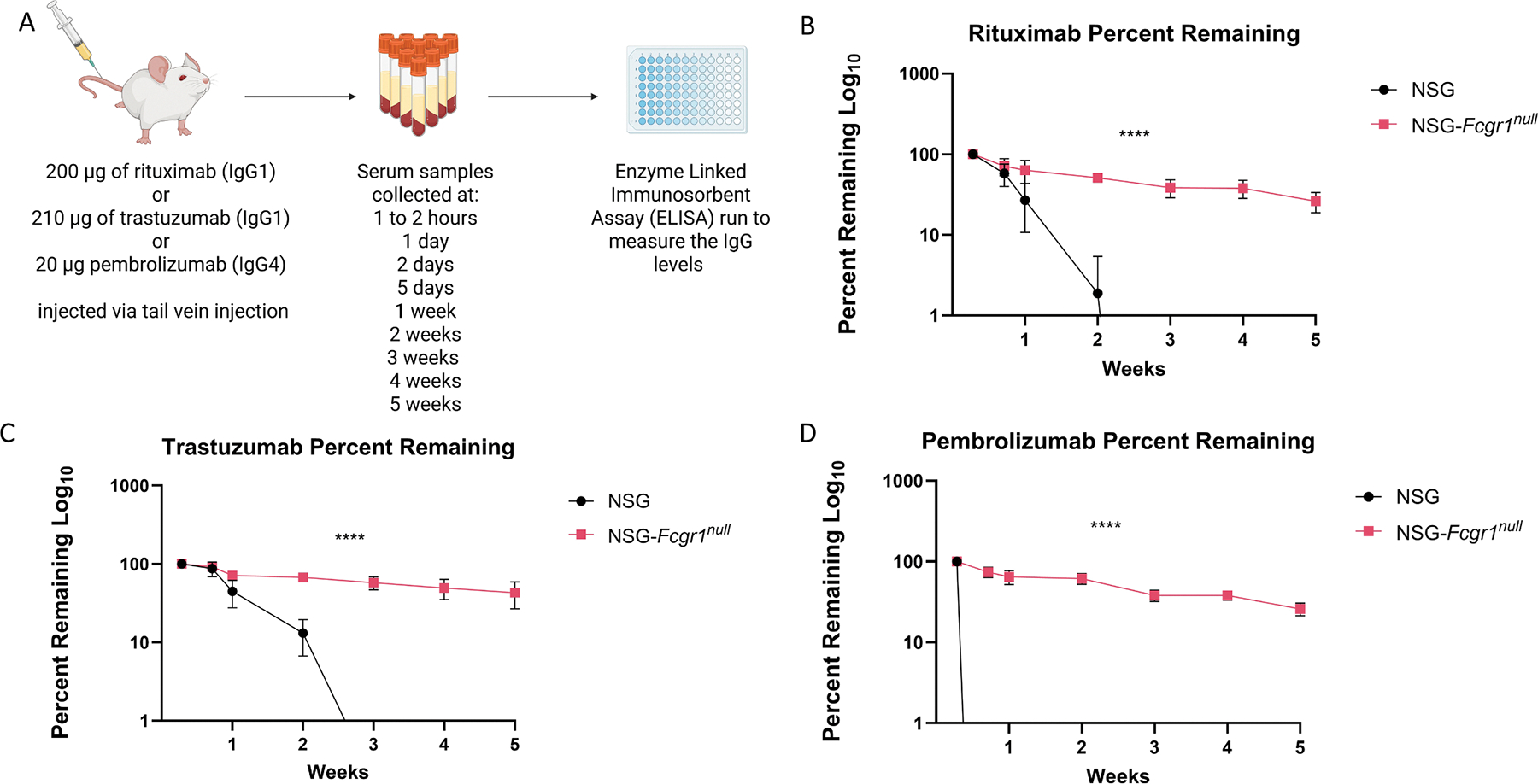
NSG-*Fcgr1*^*null*^ mice support increased IgG mAb half-lives. (A) Experimental scheme. Either 200 μg rituximab, 210 μg trastuzumab, or 20 μg pembrolizumab was injected via tail vein. Blood samples were collected at 1–2 h, 1 day, 2 days, 5 days, 1 week, 2 weeks, 3 weeks, 4 weeks, and 5 weeks. Serum IgG levels were measured by ELISA. (B-D) Serum levels of therapeutic mAbs over 5 weeks post-injection. Percent remaining calculations used the 2-day timepoint as the 100 % measure. (B) Serum levels of rituximab in 5 females and 5 males aged 5 weeks, (C) Serum levels of trastuzumab in 5 females aged 9 weeks at start. (D) Serum levels of pembrolizumab in 5 females aged 4 weeks. Significance was determined using repeated measures analysis of variance (RM-ANOVA). **** *p* < 0.0001.

Due to the gain of function mutation in the *Fcgr1* gene on NOD background strain,^30^ the NOD FcγRI protein has an increased affinity for human IgG1, IgG3 and IgG4, and NSG mice rapidly removes these IgGs from the circulation.^17,31^ To determine if the lack of FcγRI in NSG-*Fcgr1*^*null*^ mice would affect the half-life of IgG therapeutic mAbs, the clearance of three clinically utilized mAbs was measured (Fig. 2A). Injection of 200 μg of rituximab in NSG mice resulted in rapid clearance with a half-life of 2.2 ± 0.08 days. In contrast, there was an increase in half-life of approximately a 10-fold increase in the NSG-*Fcgr1*^*null*^ mice to 21.63 ± 0.80 days (Fig. 2, Table 1). Similarly, an injection of 210 μg of trastuzumab in NSG mice was followed by rapid clearance with a half-life of 4.34 ± 0.22 days compared with a half-life of 36.25 ± 4.09 days in NSG-*Fcgr1*^*null*^ mice (Fig. 2, Table 1). Finally, an injection of 20 μg of pembrolizumab in NSG mice was followed by rapid clearance with a half-life of < 1 day compared with a half-life 18.87 ± 0.49 days in NSG-*Fcgr1*^*null*^ mice. Clearance to undetectable levels in NSG mice occurred before 1 week for pembrolizumab and by 3 weeks for both rituximab and trastuzumab. In contrast, NSG-*Fcgr1*^*null*^ mice maintained detectable levels of the therapeutic mAbs throughout the five-week observation period (Fig. 2B–D). This represents a highly significant difference in the clearance of IgG therapeutic mAbs between these two strains (*p* < 0.0001, RM-ANOVA). The half-life for the therapeutic mAbs in the NSG-*Fcgr1*^*null*^ mice was significantly increased 10 to 20-fold (Table 1). The increase of the area under the curve (AUC) seen in the NSG-*Fcgr1*^*null*^ mice compared to the NSG mice indicates greater exposure of the NSG-*Fcgr1*^*null*^ mice to the therapeutic mAbs.

### HSC engraftment

**Fig. 3.**
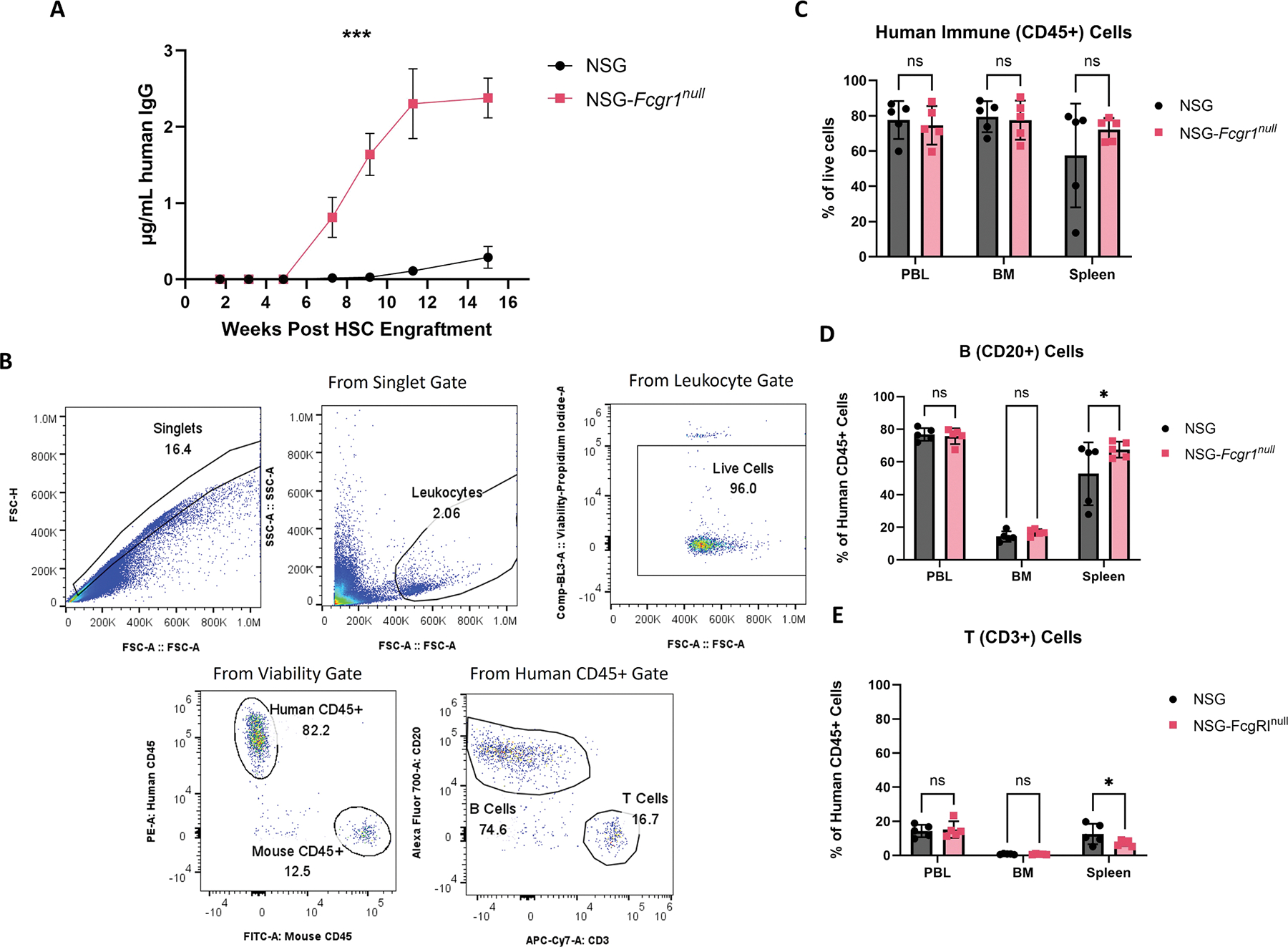
NSG-*Fcgr1*^*null*^ supports increased IgG mAb levels in HSC engrafted mice. (A) Serum levels of human IgG in 5 females for each cohort over 16 weeks post HSC transfer. Significance was determined using repeated measures analysis of variance (RM-ANOVA). *** *p* = 0.0006 (B) Representative flow cytometry of human immune cell markers in the peripheral blood leukocytes of NSG and NSG-*Fcgr1*^*null*^ mice. (C-E) Graphs showing the percentage of cells that are human CD45+ (C), human CD20+ (D), and human CD3+ (E). Significance was determined using two-way analysis of variance (two-way ANOVA). * *p* = 0.0268. PBL - peripheral blood leukocytes; BM - bone marrow.

To investigate the effect of the knockout of the gain of function *Fcgr1* null allele on human HSC engraftment and on in vivo production of IgG by human B cells, NSG and NSG-*Fcgr1*^*null*^ mice were engrafted with human HSCs from UCB. At 15 weeks-post engraftment, all recipient mice showed human CD45+ cells in the blood, bone marrow and spleen. When NSG-*Fcgr1*^*null*^ mice were engrafted with HSCs, they exhibited and maintained higher levels of circulating human IgG compared with NSG controls (Fig. 3A). At 10–14 weeks post HSC engraftment, human IgG levels were 8-fold higher NSG-*Fcgr1*^*null*^ mice. In these same mice, the percentage of human CD45+ cells and the proportion of those CD45+ cells that co-expressed human CD20 were not significantly different between the NSG and the NSG-*Fcgr1*^*null*^ groups (Fig. 3C–D). Furthermore, there was no marked difference in the percentage of human CD45+ cells that concomitantly expressed CD3 in bone marrow (BM) or peripheral blood leukocytes (PBL) between the NSG and NSG-*Fcgr1*^*null*^ mice and a slight difference in the spleen (Fig. 3E, *p* = 0.0268, two-way ANOVA).

### Keytruda functional testing

**Fig. 4.**
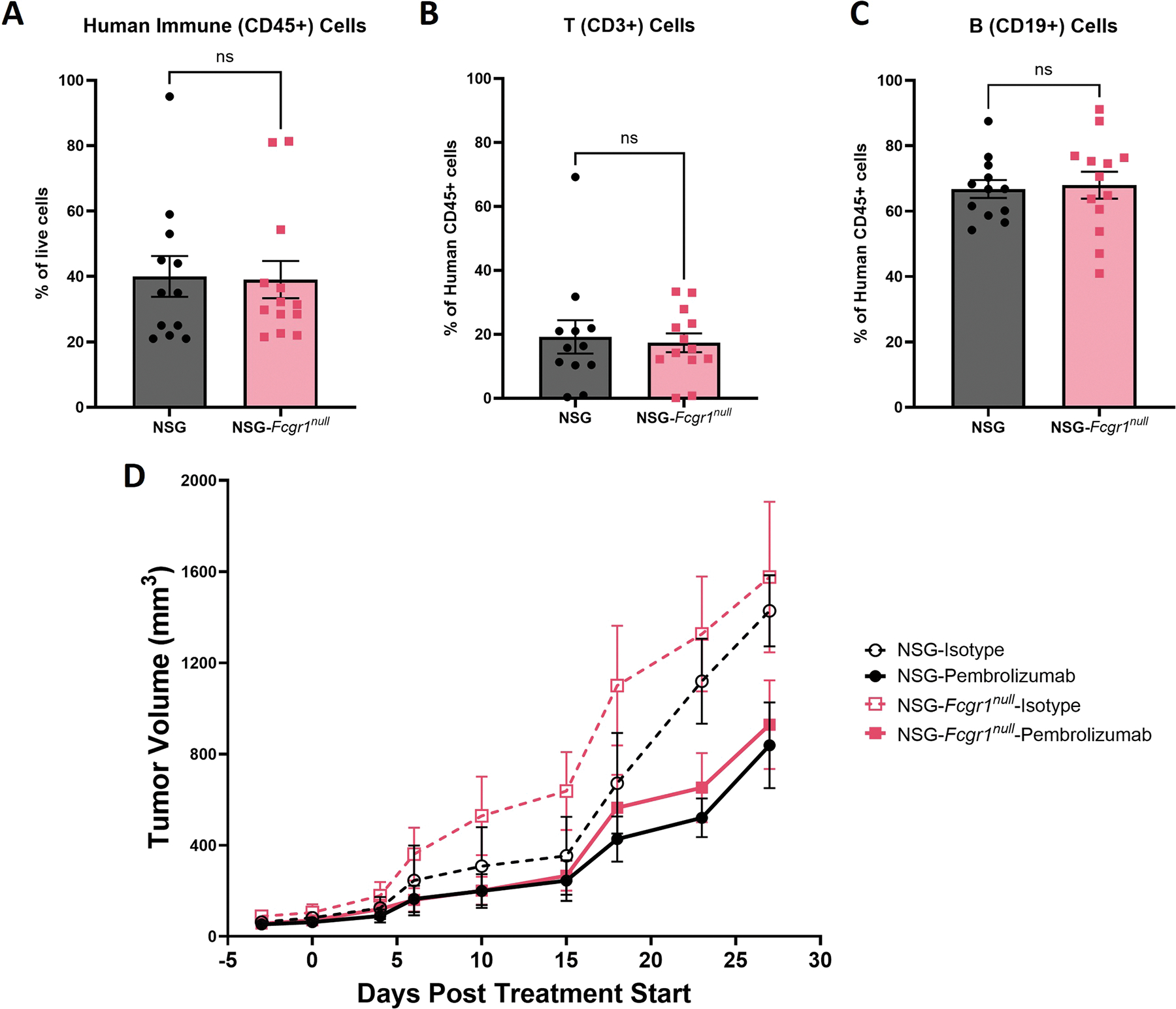
Pembrolizumab treatment efficacy in NSG-*Fcgr1*^*null*^ is not significantly different from NSG controls. (A-C) There was no significant difference (Unpaired T-test) in the percentage of human immune cells (A, *p* = 0.911), T cells (B, *p* = 0.754), or B cells (C, *p* = 0.819) in the PBL of NSG and NSG-*Fcgr1*^*null*^ 16 weeks post human HSC transfer. (D) HSC-engrafted Male and female NSG and NSG-*Fcgr1*^*null*^ were implanted with a patient-derived melanoma line by subcutaneous injection. There was no significant difference between NSG and NSG-*Fcgr1*^*null*^ tumor volume for the pembrolizumab treated groups or the isotype control groups. Each group consisted of 3–5 mice and both males and females were used. Error bars represent SEM. PBL – peripheral blood leukocytes.

To determine if the increased half-life of the therapeutic antibodies in the NSG-*Fcgr1*^*null*^ mice would translate to elevated in vivo efficacy, we engrafted NSG and NSG-*Fcgr1*^*null*^ mice with human HSC. There were no significant differences in numbers of human CD45+ cells or percentages of T cells or B cells in the peripheral blood in NSG-*Fcgr1*^*null*^ mice compared with NSG controls at 16 weeks post-injection with human HSC (Fig 4, A–C). After confirming human immune system engraftment, the mice were xenografted with patient derived melanoma cells.^21^ We found that there was a significant difference in tumor growth between the groups that received an IgG4 isotype control and the pembrolizumab (3-way ANOVA, *p* < 0.0001), but no significant difference between the strains in the treatment groups (Fig. 4D, 3-way ANOVA, *p* = 0.4154).

### Discussion

This study targeted the *Fcgr1* gene in NSG mice and investigated the effect of FCGR1 deficiency on human IgG clearance. Mouse FCGR1 binds with high affinity to human IgG1, 3, and 4^14^. Preliminary pharmacokinetic studies using three clinically relevant human IgG mAbs revealed a nine-to-twenty-four-fold increase in half-life of these therapeutic mAbs in NSG-*Fcgr1*^*null*^ mice compared with NSG mice. When compared to NSG controls, NSG-*Fcgr1*^*null*^ mice supported markedly increased half-lives of three different therapeutic antibodies, rituximab (IgG1), trastuzumab (IgG1), and pembrolizumab (IgG4). The increase in half-life was greatest for pembrolizumab, (24-fold increase). The increases in half-life were also highly significant for rituximab and trastuzumab, with both showing approximately a 9-fold increase in half-life in the NSG-*Fcgr1*^*null*^ mice when compared to the NSG controls (Table 1).

The purpose of this study is to investigate the preliminary pharmacokinetics of mAbs in this mouse model, but detailed pharmacokinetic studies are being planned. The doses for trastuzumab and pembrolizumab used in this study are based on the human dose, however the rituximab dose is much lower. Clinical doses of rituximab, trastuzumab, and pembrolizumab are the following: 375 mg/m^2^, 4–8 mg/kg for initial bolus dose, and 200 mg, respectively. Based on the Food and Drug Administration (FDA) guidelines, estimated doses are 200 ug, 210 ug, and 20 ug, respectively. When converting given doses to human dose, these are equivalent to 28 mg/m^2^ (Mouse body surface area = 0.007),^32^ 10.5 mg/kg, and ~70 mg (or 1 mg/kg). (Doses extrapolated based on mouse weight or body surface area).

There is an inverse relationship between clearance rate and dosage of drug. At higher doses these is a decreased rate of clearance.^33^ The very short half-life of pembrolizumab in NSG mice (<1 day) may be associated with the lowered dose (20 μg) injected compared to 200 μg of rituximab and trastuzumab injected. To interrogate receptor involvement and non-linear pharmacokinetics, future studies will include dose dependent measurements. Furthermore, these studies will include longer experimental timelines and allometric scaling to further support the comparison to human clearance.^34^ Additionally, we found that following the engraftment of human CD34+ HSCs, NSG-*Fcgr1*^*null*^ mice showed similar levels of human immune system development as compared to NSG mice, but HSC engrafted NSG-*Fcgr1*^*null*^ mice had increased concentrations of human IgG in serum compared to NSG controls. This difference is not due to a disparity in the levels of human immune or human B cells present in the mice and is consistent with delayed clearance of human IgG in NSG-*Fcgr1*^*null*^ mice.

It has been shown that an increase in antibody half-life correlates to improved in vivo efficacy.^35^ When testing if the increased half-life of the therapeutic mAbs correlated with increased efficacy in a PDX model, we found that there was no significant difference on effects of pembrolizumab treatment of a PDX melanoma between the NSG-*Fcgr1*^*null*^ mice and the NSG controls. This may be associated with the dosing schedule used. The dose used in our study was determined from a previous experiment with NSG mice.^36^ It is known from studies in humans that there is a point in which an increased dose of pembrolizumab no longer confers increased efficacy.^37–39^ This suggests the possibility that while our NSG-*Fcgr1*^*null*^ mice have an increased pembrolizumab concentration in the plasma, this may not lead to increased anti-tumor properties. Future studies to determine a dose that does not saturate the system are planned.

Human IgG1, IgG2, and IgG4 measured in the clinic all have half-lives of approximately 22 days, while IgG3 has a half-life of approximately 7 days potentially due to less efficient recycling of IgG3 by FcRn^13,40^; Human IgG1 has a high affinity to all FcγRs second only to IgG3. IgG3 is not a good candidate for mAbs as antibodies of this subtype are less stable and more prone to causing immune responses in patients.^13^ IgG2 has high affinity only for FcγRIIa while IgG4 only has high affinity for FcγRI.^41^ The majority of human therapeutic mAbs are IgG1 or IgG4 isotype.^16^

Human IgG1 has been found to be the most potent activator of mouse effector cells and interacts with them similarly to humans, making mice promising preclinical models for testing human IgG1 mAbs.^42^ IgG1 has been the primary subclass used in antitumor therapeutic mAbs due to its strong activation of antibody-dependent cell-mediated cytotoxicity (ADCC) and antibody-dependent cellular phagocytosis (ADCP). These mechanisms alone can lead to tumor death, but can also be combined with other mechanisms, such as the blocking of PDL-1 to prevent T cell dysfunction for more effective treatment. Both rituximab and trastuzumab are IgG1 isotypes which augments their direct killing functions through ADCC and ADCP.^41^

Rituximab is a chimeric mAb targeting CD20. It incorporates human IgG1κ constant regions with mouse variable regions.^43^ It is mainly used to treat B-cell non-Hodgkin’s lymphoma (NHL) in addition to other cancers such as hairy cell leukemia and chronic lymphocytic leukemia (CLL).^44,45^ Rituximab kills CD20+ *B* cells through four different mechanisms: direct killing, complement-dependent cytotoxicity (CDC), ADCC, and ADCP.^44^ Additionally, rituximab has been used to treat some autoimmune diseases including systemic lupus erythematosus (SLE) and rheumatoid arthritis (RA) by depleting B cells, thereby lowering production of autoantibodies.^3^ Depending on the disease being treated, the dose and treatment schedule for rituximab therapy varies. For NHL and CLL, the recommended treatment schedule is 375 mg/m^2^ once weekly or on the first day of chemotherapy cycles. In the treatment of RA, two 1000 mg intravenous infusions are given 2 weeks apart. The half-life varies based on the treatment schedule and dose, but it ranges from 18–32 days. This is similar to the rituximab half-life of 21.6 days in NSG-*Fcgr1*^*null*^ mice.

Trastuzumab is a humanized recombinant IgG1 antibody with murine complementarity-determining regions grafted onto a human framework.^46^ It binds to an extracellular domain of HER2 and is used to treat HER2+ breast and gastric cancers.^47^ The binding inhibits HER2 homodimerization, thus preventing signaling.^48^ Additionally, there is some cytotoxic function by ADCC mediated through Fc receptor binding on NK cells, dendritic cells, and macrophages.^49^ Trastuzumab is typically either combined with a chemotherapy regimen or conjugated with a drug.^50^ Treatment consists of a 4 mg/kg loading dose and then weekly maintenance doses of 2 mg/kg given either intravenously or subcutaneously.^51,52^ The reported clinical half-life in humans is 28 days^53^ which approximates the half-life of 35.6 days in NSG-*Fcgr1*^*null*^ mice compared with the half-life of 4.2 days in NSG mice.

Pembrolizumab is a humanized IgG4κ isotype that targets PD-1.^54^ PD-1 is a receptor on T cells, that when bound to programmed death ligand 1 (PD-L1) expressed by a target cell, reduces cytotoxic T cell function. IgG4 was selected as the isotype for pembrolizumab as it only binds to FcγRI with high affinity and does not produce strong effector functions against the target. This is important as the blocking of PD-1 on T cells prevents tumor cells from utilizing the PD-L1/PD-1 axis as a means of non-detection. If effector cells were engaged against these T cells, as may happen if it was an IgG1 isotype antibody, they could be depleted,^41^ making them unable to kill the tumor cells. Pembrolizumab treats multiple types of cancer, including melanoma, non-small-cell lung cancer, and renal cell carcinoma. For use in adult patients with cancer, the dose recommended in the package insert is 200 mg every three weeks, or 400 mg every 6 weeks. Data from the manufacturer indicates that the half-life is 22 days; this was determined using 2993 patients who had varying cancers and were given doses between 1 and 10 mg/kg every 2 weeks, 2 to 10 mg/kg every 3 weeks, or 200 mg every 3 weeks compared with a half-life of 18.9 days in NSG-*Fcgr1*^*null*^ mice. IgG4 is more commonly used when a target is on an immune cell, such as PD-1, the target of pembrolizumab.^41^ Preliminary efficacy studies were carried out only for pembrolizumab. Future expanded efficacy studies are needed.

Previously, the *Fcgr1* gene was inactivated in C57BL/6–129 chimeric mice. In studies utilizing these mice, it was found that FcγRI is the most effective Fc receptor for endocytosing soluble IgG, and that it is the main receptor involved in this process. Once endocytosed, IgG can either be broken down through lysosomal degradation or recycled back into circulation if bound to FcRn.^55^ For therapeutic antibodies to function, they need to bind to the target receptor and remain on the cell surface so that it can be recognized by effector cells for ADCC to occur. Preventing IgG endocytosis has been shown to be an effective adjuvant for ADCC-mediating mAbs.^56^ IgG endocytosis still occurs in the absence of FcγRI most likely due to the presence of FcγRIII which has similar uptake abilities.^57^ It has been found in some tumor studies with these mice that FcγRI can be essential for tumor killing through ADCC, while others have found no difference.

A previous report described decreased FcγRI activity following targeting the *Fcer1g* gene.^58^ The Fc receptor γ chain is a component of several FcRs.^28^ It was initially believed that the *Fcer1g* gene mutation caused loss of expression of FcγRI, and FcγRIII, as well as FcεRI. However, further research has showed that targeting *Fcer1g* leads to a loss of expression of FcγRIII, FcγRIV and FcεRI and a decrease but not complete expression of FcγRI.^57,59,60^

The *Fcer1g* knockout was originally carried out in C57BL/6–129 chimeric mice but has been backcrossed to NOD.Cg-*Prkdc*^*scid*^ *Il2rg*^*tm1Sug*^/JicTac (NOG) mice. NOG mice, similar to NSG mice, are severely immunodeficient, lacking adaptive immune responses and impaired innate immune responses. The *Fcer1g* mutation has been combined with inactivation of the *Fcgr2b* gene which inactivates FcγRIIb in mice. Overall, these mice, NOD.Cg-*Fcgr2b*^*tm1Ttk*^ *Fcer1g*^*tm1Rav*^ *Prkdc*^*scid*^ *Il2rg*^*tm1Sug*^ (NOG-FcγR^−/−^) lack mouse Fc receptors FcγRIII, FcγRIV, FcγRIIb, and FcεRI and have a decreased expression of FcγRI. These mice, however, exhibit rapid mAb clearance, potentially due to the continued expression of FcγRI.^61^

Thaller et al. (2023)^62^ inactivated the *Fcer1g* gene in BALB/c *Rag2*^*tm1Fwa*^ *Il2rg*^*tm1Cgn*^ *Sirpa*^*NOD*^ Tg(HLA-A/H2-DB2M)^1Bpe^, Tg (HLA-DRB1×1501)^#Lfug^ (BRGSA2DR2) mice to investigate the role of human and mouse FcR-γ_+_ cells as well as complement in mouse models in therapeutic mAb testing. Overall, they found that mouse complement as well as mouse and human FcR-γ_+_ cells all play a role in the depletion of B cells by Rituximab-variants, suggesting that restoration of complement function should increase rituximab activity.^63^

Direct interaction with Fc receptors can affect the function of mAbs. This is generally through the initiation of effector functions such as CDC, ADCC, or ADCP.^64^ FcγRI specifically has been shown to play a role in ADCP by activating myeloid cells to phagocytose cells.^60^ Therefore, it is possible that our NSG-*Fcgr1*^*null*^ mice may have limited effector function. In order to overcome this limitation, we are investigating creating a “knock in” model, where human *FCGR1* cDNA has been knocked into the mouse *Fcgr1* locus. This should disrupt the gain of function *Fcgr1* d allele that causes the increased clearance of IgGs while preserving translatable physiological clearance levels for drug development.

In April 2025, the food and drug administration (FDA) released its “Roadmap to Reducing Animal Testing in Preclinical Safety Studies”. This communication described how the FDA is planning to reduce the number of animals used in drug development, specifically highlighting mAbs. Their stated goal is to make drug development “more ethical, more efficient, and more predictive of human outcomes”. This roadmap included the support of new approach methodologies (NAMs), including in vitro systems such as organs-on-chips as well as large-scale computer modelling. Furthermore, in this roadmap, they highlight that “humanized transgenic [mice] can reduce animal numbers and pain”.^65^ Our model described here can be used in accordance with these new FDA guidelines to decrease the use of animals in drug development by increasing the translatability of the mice models used.

With this model showing an increased PK profile for IgG1 and IgG4-based mAbs, there is a high likelihood that NSG-*Fcgr1*^*null*^ mouse model could be used to evaluate the pharmacokinetics of other mAb-based therapies. Many mAb-based therapies exist, including antibody-drug conjugates (ADCs) and bispecific antibodies. ADCs are a class of antibody in which a payload is conjugated to a mAb using a linker to allow a targeted release of the payload. These payloads typically involve chemotherapies bound to a tumor-targeting mAb, allowing specific tumor cell killing with limited off-target toxicity.^66^ This class of therapeutics is often tested in NSG mice to study their impact on tumors,^17,67,68^ but their efficacy has shown to be impacted because of the increased clearance of ADC in NSG mice.^17^ In addition, bispecific antibodies, antibodies that are able to simultaneously bind to two distinct antigens, are another therapy that has been tested using NSG mice.^69–71^ Although no pharmacokinetic study has been performed in NSG mice and compared to multiple mouse strains of a bispecific antibody, IgG-based bispecific antibodies will interact with FcR due to the Fc domain. As a result, it may have an increased clearance due to the gain of function mutation of NSG mice and could benefit from the NSG-*Fcgr1*^*null*^ mouse model. In summary, we present the first description of a novel NSG model that greatly enhances the longevity of human IgG1 and IgG4 mAbs. Future clearance and efficacy studies are required to expand on these findings.

## Supplementary Material

Supplemental Figure 1

Supplementary material associated with this article can be found, in the online version, at doi:10.1016/j.xphs.2025.103964.

## Figures and Tables

**Table 1:** Pharmacokinetic parameters of therapeutic IgG mAbs in NSG and NSG-*Fcgr1^null^* mice.

		Rituximab	Trastuzumab	Pembrolizumab

**NSG**	Dose injected (μg)	200	210	20
	AUC_Inf_ (days*μg/mL)	498.2 (30.6)	2204.0 (57.7)	24.59 (1.09)
	Lambda.z (1/days)	0.346 (0.011)	0.166 (0.010)	0.907 (0.022)
	t_1/2_ (days)	2.21 (0.08)	4.34 (0.22)	0.772 (0.018)
	Vd (mL)	1.70 (0.10)	0.609 (0.043)	0.929 (0.032)
	CL (mL/day)	0.539 (0.028)	0.0961 (0.0027)	0.840 (0.030)
**NSG-*Fcgr1^nul1^***	Dose (μg)	200	210	20
	AUC_Inf_ (days*μg/mL)	2494.6 (101.0)	7790.1 (709.8)	215.84 (5.61)
	Lambda.z (1/days)	0.035 (0.001)	0.025 (0.005)	0.037 (0.001)
	t_1/2_ (days)	21.63 (0.80)	36.25 (4.09)	18.87 (0.491)
	Vd (mL)	2.68 (0.12)	1.362 (0.073)	2.539 (0.064)
	CL (mL/day)	0.090 (0.004)	0.031 (0.004)	0.094 (0.002)
***p* Values**	AUC_Inf_ (days*μg/mL)	0.0001	0.0288	0.0001
	Lambda.z (1/days)	< 0.0001	0.0043	0.0001
	t_1/2_ (days)	< 0.0001	0.0121	0.0001
	Vd (mL)	0.0566	0.0071	0.0001
	CL (mL/day)	0.0007	0.0011	0.0003

Standard error of the mean (SEM) is in parentheses. p values were determined by student’s t-test. AUC_Inf_ - Area under the curve to infinity; Lambda.z - apparent terminal rate constant; Vd - Volume of Distribution; CL - Clearance.

## Data Availability

Data is available upon request.

## Abbreviations:

mAbs: Monoclonal antibodies
HSCs: hematopoietic stem cells
NSG: NOD.Cg-Prkdc^scid^ Il2rg^m1Wjl^/SzJ
NK: natural killer
Sirpa: signal regulatory alpha
PBMC: peripheral blood mononuclear cell
PCR: polymerase chain reaction
PD-1: programmed death protein-1
TMB: tetramethylbenzidine dihydrochloride
UCB: umbilical cord blood
FcγRs: Fc gamma receptors
BM: bone marrow
PBL: peripheral blood leukocytes
FDA: Food and Drug Administration
mFcγRI: Mouse FcγRI
ADCC: antibody-dependent cell-mediated cytotoxicity
ADCPP: antibody-dependent cellular phagocytosis
NHL: Non-Hodgkin’s lymphoma
CLL: chronic lymphocytic leukemia
CDC: complement-dependent cytotoxicity
SLE: systemic lupus erythematosus
HER2: human epidermal growth factor receptor 2
PD-L1: programmed death protein ligand 1
RA: rheumatoid arthritis
ELISA: enzyme-linked immunosorbent assay
PDX melanoma: patient derived xenograft melanoma
FDA: Food and Drug Administration
NAMs: new approach methodologies
