# Supplementary material for: Increased longevity of circulating human IgG in an NSG Fc gamma receptor-1 deficient humanized mouse model
Source: J Pharm Sci. Author manuscript; Available in PMC 2025 Dec 9. (PMC12687882; doi:10.1016/j.xphs.2025.103964)
Supplement: Supplemental Figure 1 [file NIHMS2120742-supplement-Supplemental_Figure_1.pdf]

Supplemental Figures

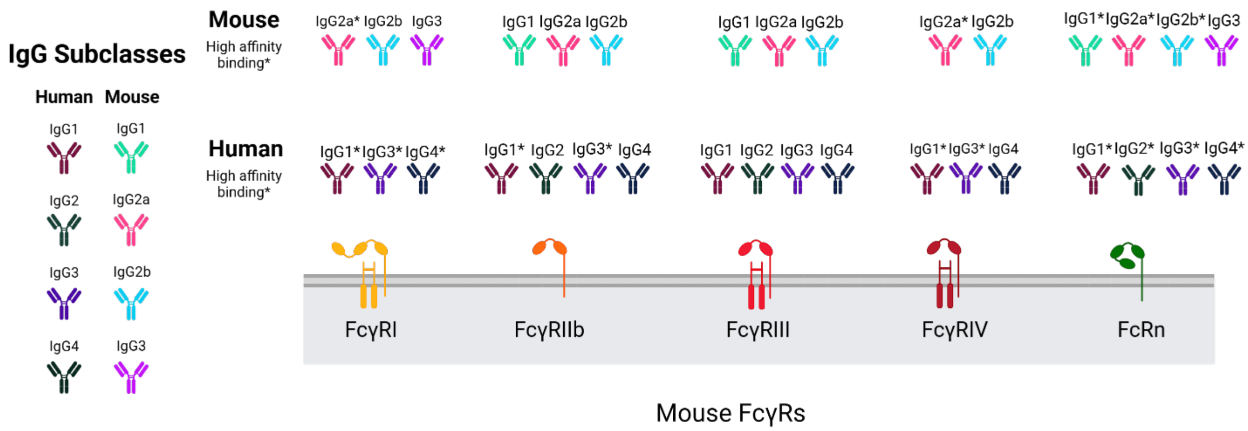

Supplemental Figure 1. Illustration of Human and Mouse IgG subclasses that indicates specificity of high affinity binding to human and mouse Fc receptors. Those that bind with high affinity are indicated with an asterisk [5,34]. Image made with BioRender.
